# Noninfectious mixed cryoglobulinaemic glomerulonephritis and monoclonal gammopathy of undetermined significance: a coincidental association?

**DOI:** 10.1186/s12882-020-01941-3

**Published:** 2020-07-23

**Authors:** Adam L. Flavell, Robert O. Fullinfaw, Edward R. Smith, Stephen G. Holt, Moira J. Finlay, Thomas D. Barbour

**Affiliations:** 1 Department of Nephrology, The Royal Melbourne Hospital, Parkville, Australia; 2 Department of Chemical Pathology, The Royal Melbourne Hospital, Parkville, Australia; 3 Department of Medicine, The University of Melbourne, Melbourne, Australia; 4 Department of Anatomical Pathology, The Royal Melbourne Hospital, Parkville, Australia

**Keywords:** Cryoglobulinaemia, Monoclonal gammopathy of renal significance, Glomerulonephritis

## Abstract

**Background:**

Cryoglobulins are cold-precipitable immunoglobulins that may cause systemic vasculitis including cryoglobulinaemic glomerulonephritis (CGN). Type 1 cryoglobulins consist of isolated monoclonal immunoglobulin (mIg), whereas mixed cryoglobulins are typically immune complexes comprising either monoclonal (type 2) or polyclonal (type 3) Ig with rheumatoid activity against polyclonal IgG. Only CGN related to type 1 cryoglobulins has been clearly associated with monoclonal gammopathy of undetermined significance (MGUS) using the conventional serum-, urine- or tissue-based methods of paraprotein detection.

**Case presentation:**

We present four patients with noninfectious mixed (type 2 or 3) CGN and MGUS. Two patients had type 2 cryoglobulinaemia, one had type 3 cryoglobulinaemia, and one lacked definitive typing of the serum cryoprecipitate. The serum monoclonal band was IgM-κ in all four cases. Treatments included corticosteroids, cyclophosphamide, plasma exchange, and rituximab. At median 3.5 years’ follow-up, no patient had developed a haematological malignancy or advanced chronic kidney disease. Other potential causes of mixed cryoglobulinaemia were also present in our cohort, notably primary Sjögren’s syndrome in three cases.

**Conclusion:**

Our study raises questions regarding the current designation of type 2 CGN as a monoclonal gammopathy of renal significance, and the role of clonally directed therapies for noninfectious mixed CGN outside the setting of haematological malignancy.

## Background

Cryoglobulinaemia is defined by the presence of circulating immunoglobulin (Ig) that aggregates in vitro at temperatures < 37 °C, and redissolves on rewarming [1]. For cryoglobulinaemia to be detected, blood sampling, clotting and serum separation by centrifugation must ideally be performed at 37 °C, before serum storage at 4 °C for up to 7 days [2]. Any significant cryoprecipitate (usually > 0.05 g/L or cryocrit > 1%) may then be quantified and analyzed by electrophoresis and immunofixation after washing and redissolving at 37 °C. The classification system for cryoglobulinaemia devised by Brouet and colleagues distinguishes three main types [3]. Type 1 cryoglobulins consist of monoclonal Ig (mIg) or biclonal Ig, and occur in patients with clonal B cell or plasma cell disorders [4]. So-called ‘mixed’ cryoglobulins are considered as immune complexes typically comprising either monoclonal (type 2) or polyclonal (type 3) Ig (mostly IgM) with rheumatoid factor activity against the Fc portion of polyclonal IgG. Infections are the commonest cause of mixed cryoglobulinaemia, notably hepatitis C virus (HCV) [5] and hepatitis B virus (HBV) [6], together with human immunodeficiency virus (HIV) and numerous other viral, bacterial, parasitic and fungal infections [1, 7]. Noninfectious causes of mixed cryoglobulinaemia include autoimmune diseases, especially primary Sjögren’s syndrome (pSS) [8], and the malignant clonal disorders [9].

There is some uncertainty as to whether cryoglobulins are truly pathogenic in vivo, given that disease manifestations including systemic vasculitis occur in only a minority of patients with detectable cryoglobulinaemia [10]. The systemic vasculitis of cryoglobulinaemia is exemplified by cryoglobulinaemic glomerulonephritis (CGN), which can be classified as type 1 or mixed according to which type of cryoglobulin is found in association. Classic renal histological features of CGN, including membranoproliferative glomerulonephritis (MPGN), intracapillary ‘pseudothrombi’, crescents and small vessel vasculitis are fairly nonspecific [11]. On the other hand, the impression that renal causation is directly attributable to glomerular deposition of cryoglobulins may be strengthened by electron microscopy (EM) showing curvilinear microtubules, suggestive of aggregated cryoglobulins [12, 13], or immunohistochemistry showing light chain restriction of pseudothrombi in the case of type 1 CGN [14, 15].

Monoclonal gammopathy is diagnosed when mIg secreted into the circulation by a proliferating clone of plasma cells or B cells is detected by means of serum protein electrophoresis (SPEP), immunofixation (SIFE) or free light chain assays (SFLC), or urine protein electrophoresis (UPEP) or immunofixation (UIFE) [16]. Further evaluation is often required for one or other of the malignant clonal disorders, which include multiple myeloma, Waldenström’s macroglobulinaemia, B cell lymphoma and chronic lymphocytic leukemia. However, in most patients, monoclonal gammopathy of undetermined significance (MGUS) or another pre-malignant condition is diagnosed. Previous studies have established a clear association of type 1 CGN not only with malignant clonal disorders, but also with MGUS [14, 17–20]. This has led to the inclusion of type 1 CGN within the disease classification of monoclonal gammopathy of renal significance (MGRS) [21–23]. This term recognizes that certain renal lesions may be the result of nephrotoxic mIg produced by small (i.e. pre-malignant) plasma cell or B cell clones, with confirmation in many cases based on light chain restricted renal staining [24].

We undertook this study in our patient cohort to assess whether mixed (type 2 or 3) CGN is also sometimes diagnosed in patients with MGUS, as noted above for type 1 CGN. Our investigation was prompted by the surprising inclusion of type 2 CGN within the MGRS disease classification [21–23], despite having only a very weak association with MGUS in the published medical literature, and the inability to confirm light chain restriction of renal tissue owing to the polyclonal Ig component of type 2 cryoglobulins [11]. We included cases for the period 2002–2019 in which renal histology was compatible with CGN, with cryoglobulinaemia (> 0.05 g/L) and the detection of a serum monoclonal band based on SPEP/SIFE. Patients were excluded where type 1 cryoglobulinaemia was confirmed on immunofixation of the cryoprecipitate and/or light chain restricted staining (as assessed by immunofluorescent staining of paraffin-embedded tissue after protease digestion (paraffin-IF) [25]). Patients with a malignant clonal disorder were also excluded (with a minimum requirement in our study for bone marrow aspirate and trephine (BMAT) with flow cytometry of the aspirate and/or peripheral blood, and computed tomography with positron emission tomography (CT-PET)). Finally, patients with a potential infectious aetiology for mixed cryoglobulinaemia were excluded (with a minimum requirement for blood cultures, HBV surface antigen, and HBV core, HCV, and HIV antibodies), as were those in whom vasculitis was potentially due to recognised causes other than cryoglobulinaemia (based on serologies comprising at a minimum anti-neutrophil cytoplasmic, anti-glomerular basement membrane, anti-double stranded DNA, anti-U_1_-ribonucleoprotein and anti-cardiolipin antibodies, and lupus anticoagulant).

## Case presentation

Of twenty patients at our hospital with noninfectious mixed CGN in whom SPEP/SIFE was performed, we present four cases with confirmed MGUS, including three females and one male (Table 1). Median age at presentation was 54 years (range 47–66 years). All patients had microscopic haematuria and proteinuria, with a median urinary protein creatinine ratio (uPCR) of 106 mg/mmol (range 70-134 mg/mmol). Renal function was significantly impaired in three of the four patients, with a median serum creatinine overall of 221 μmol/L (range 80-292 μmol/L) and eGFR of 27 mL/min per 1.73m^2^ (range 15-70 mL/min per 1.73m^2^, MDRD). All patients had mildly reduced serum albumin levels, and serum complement C4 ± C3 levels were low in three patients. All three female patients had previously been diagnosed with pSS and showed seropositivity for antinuclear, SSA/Ro and SSB/La antibodies. In the weeks following diagnosis of CGN, Patient 2 underwent laparotomy for an incidental finding of cholangiocarcinoma.

**Table 1:** Demographic and renal clinical features at time of renal biopsy

Patient	Age (Years)	Sex	Extrarenal clinical features	Comorbid conditions	Urine studies			Serum studies					
					Red cells	PCR (< 30 mg/mmol)	ACR (< 3.5 mg/mmol)	Creatinine (μmol/L)	eGFR (mL/min per 1.73 m^2^)^a^	Albumin (3.5-5 g/dL)	C3 (0.9–1.8 g/L)	C4 (0.16–0.5 g/L)	ANA/SSA/SSB
1	47	F	Purpura, arthritis	pSS	Pos	70	33	256	18	2.6	0.46	< 0.03	Pos
2	60	F	Purpura, benign lymphadenopathy	pSS, CC	Pos	126	75	292	15	3.2	1.1	0.27	Pos
3	66	M	–	–	Pos	86	55	186	35	2.7	0.76	< 0.03	Neg
4	47	F	Purpura, arthritis	pSS, hypoGG	Pos	134	–	80	70	3.1	1.27	< 0.03	Pos

*PCR* protein creatinine ratio; *ACR* albumin creatinine ratio; *eGFR* estimated glomerular filtration rate; *ANA* antinuclear antibody; *SSA* anti-Ro; *SSB* anti-La; *pSS* primary Sjögren’s syndrome; *CC* cholangiocarcinoma; *hypoGG* hypogammaglobulinaemia

^a^ Modified diet in renal disease (MDRD)

**Fig. 1:**
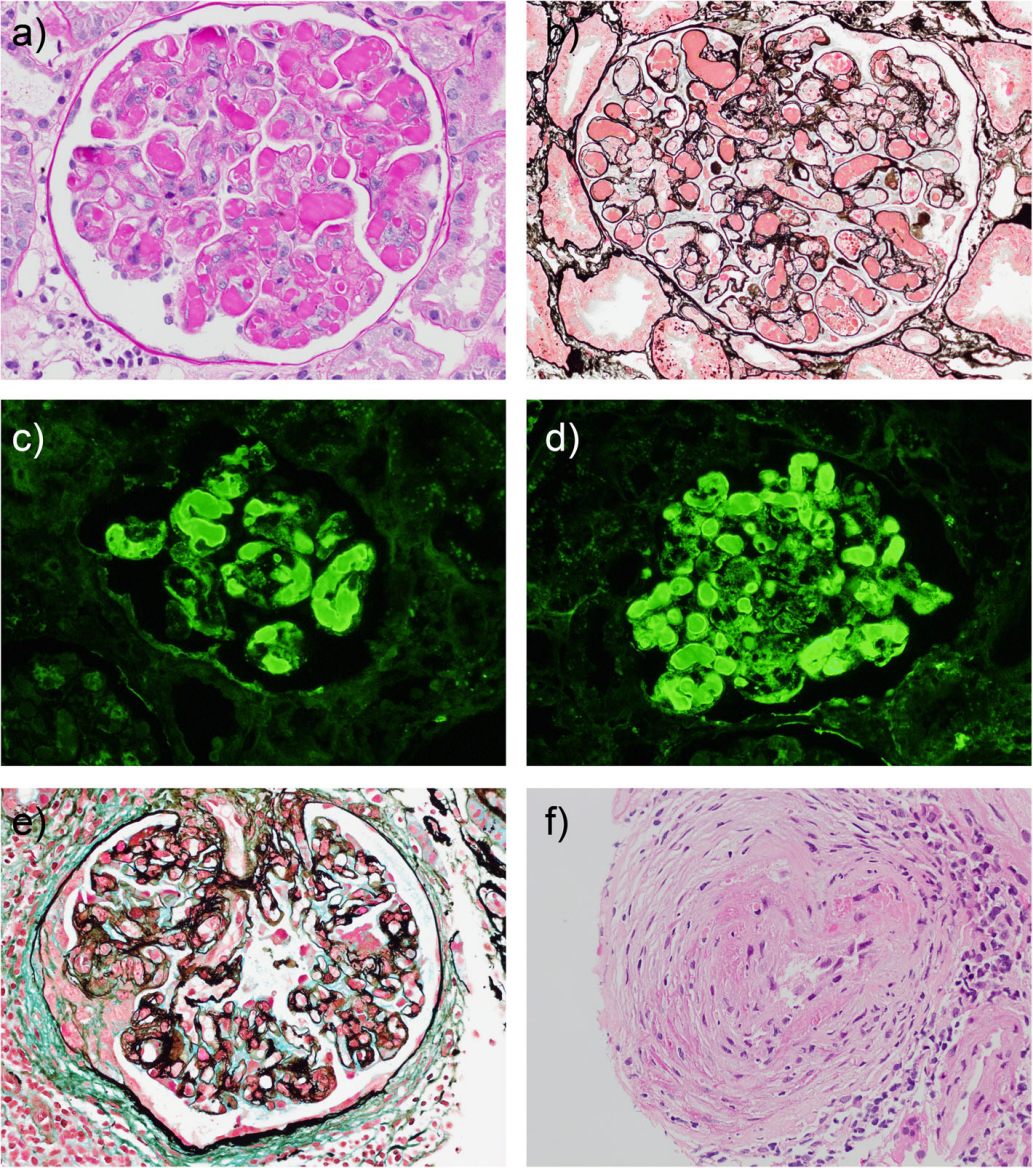
Histology. Light microscopy in patient 1 with **a** periodic acid-Schiff (PAS) stain and **b** silver stain showing MPGN with double contours and striking intraluminal, PAS-positive pseudothrombi. Equal (+++) intensity of paraffin-IF staining of pseudothrombi for **c** κ and **d** λ light chain. In patient 2, **e** silver stain showing a small cellular crescent with necrosis, and **f** haematoxylin and eosin stain of a small artery with concentric intimal arteritis. Magnification ×40

Renal biopsy revealed histological features of CGN in all four patients (Fig. 1 and Table 2). These included MPGN in three patients, cellular crescents with arteriolar necrosis and thrombosis in one patient, and intracapillary pseudothrombi in three patients. Interstitial fibrosis ≥25% with mild glomerulosclerosis was also present in three cases. Immunohistochemistry showed variable IgG, IgM and C3 staining in capillary loops and the mesangium, with IgM and/or IgG staining of pseudothrombi in two cases. No case showed light chain restriction on paraffin-IF. EM was performed in three cases, revealing intracapillary curvilinear deposits in one case and unstructured glomerular deposits in the other two cases.

**Table 2:** Renal histology

Patient	N^**o**^ glomeruli (N^**o**^ globally sclerosed)	Pattern of glomerular inflammation	Arteriolar necrosis, thrombosis	Interstitial inflammatory infiltrate	Interstitial fibrosis (< 5%)	Pseudothrombi	Glomerular IHC (paraffin-IF)	Deposits on EM
1	25 (0)	Early MPGN	No	Patchy, mild	< 5%	Yes	Loop focal IgM+ (κ-, λ-) Pseudothrombi IgG+, IgM+++ (κ+++, λ+++)	Unstructured
2	18 (6)	Crescents (5 cellular, 1 fibro-cellular, 2 fibrous)	Yes	Light, chronic	40%	No	Mesangial focal IgM++, C3++ (κ+, λ+)	–
3	20 (5)	MPGN, endocapillary (CD68+)	No	Light, chronic	25%	Yes	Loop and pseudothrombi IgG++, IgM+++, IgA+, C3+++, C1q++ (κ ++, λ ++)	Unstructured
4	18 (2)	MPGN	No	Moderate	25%	Sparse	Mesangial and loop IgG+, IgM++, IgA+, C3++ (κ -, λ -)	Fine curvilinear

*MPGN* membranoproliferative glomerulonephritis; *IHC* immunohistochemistry; *IF* immunofluorescence; *IF* electron microscopy; *κ* kappa; *λ* lambda

Serum biochemistry at presentation (Table 3) included a median cryoglobulin concentration of 0.43 g/L (range 0.1–0.62 g/L) in three cases, with a cryocrit of 9% in the fourth case. Immunofixation of the cryoprecipitate confirmed type 2 cryoglobulinaemia with a monoclonal IgM-κ component in two patients and type 3 cryoglobulinaemia in one patient, and was not performed in the remaining patient. SPEP revealed generally small monoclonal bands of median concentration < 1 g/L (range < 1 - 2 g/L). In all four cases, the paraprotein was IgM-κ, with an IgG-κ paraprotein also present in one case (Patient 2). No patient showed bone marrow evidence of a malignant plasma cell or B cell disorder (Table 4).

**Table 3:** Biochemistry at time of renal biopsy

Patient	Serum cryoglobulin			Paraprotein						Peripheral blood flow cytometry
	Concentration (g/L)	Type	RF	SPEP (g/L)	SIFE	SFLC^a^			Spot UPEP/UIFE	
						κ (3.3–19.4 mg/L)	λ (5.7–26.3 mg/L)	κ /λ (0.26–1.65)		
1	0.1	2 or 3	Neg	2	IgM-κ	26.6	5.5	4.84	Neg	Normal
2	0.43	2 (mIgM-κ + pIgG)	Pos	< 1	IgM-κ, IgG-κ	231.9	65.3	3.55	Neg	Normal
3	0.62	3 (pIgM + pIgG)	Pos	< 1	IgM-κ	49.0	27.5	1.78	Neg	Not assessed
4	9% cryocrit	2 (mIgM-κ + pIgG)	Pos	< 1	IgM-κ	157.0	17.4	9.02	IgM-κ	Normal

*RF* rheumatoid factor; *SPEP* serum protein electrophoresis; *SIFE* serum immunofixation; *SFLC* serum free light chains; *κ* kappa; *λ* lambda; *UPEP/UIFE* urine protein electrophoresis/immunofixation; *mIg* monoclonal immunoglobulin; *pIg* polyclonal immunoglobulin

^a^Freelite assay, The Binding Site Group, Birmingham, UK

**Table 4:** Bone marrow aspirate and trephine

Patient	Trephine			Aspirate	
	Cellularity	Immunohistochemistry		Lymphoid cells (5–20%)	Flow cytometry
		Plasma cells (< 5%)	Lymphoid aggregates		
1	Normal	< 5%	One small	16%	Normal
2	Normal	< 5%	Two small	9%	Normal
3	Mildly hypercellular	< 5%	None	9%	Normal
4	Normal	< 5%	None	5%	Not assessed

Treatment consisted of corticosteroids in all patients, cyclophosphamide in three patients, plasma exchange in three patients, and rituximab in two patients, one of whom (Patient 4) later received maintenance therapy for a diagnosis of seronegative lupus nephritis with mycophenolate mofetil (Table 5). At a median follow-up of 3.5 years (range 1.5–11 years), no patient had developed a malignant clonal disorder or end-stage renal failure, or was deceased. Renal parameters were improved in all cases but one (Patient 4), with a median uPCR at last follow-up of 103 mg/mmol (range 24 – 302 mg/mmol), serum creatinine of 133 μmol/L (range 62 - 168 μmol/L), and eGFR of 45 mL/min per 1.73m^2^ (range 30-90 mL/min per 1.73m^2^). Serum C4 remained undetectable in two patients. Cryoglobulinaemia was undetectable at last follow up in all four patients, and SPEP/UPEP was negative. However, SIFE revealed an IgM-κ paraprotein (< 1 g/L) in one patient, and the SFLC ratio was elevated at 2.09 in one other patient, with no evidence for persisting monoclonal gammopathy in the remaining two patients.

**Table 5:** Treatment and last follow-up

Patient	Treatment received	Recurrent vasculitis	Urine PCR (< 30 mg/mmol)	Serum creatinine (μmol/L)	eGFR^a^ (mL/min per 1.73 m^2^)	Serum cryoglobulin	C3 (0.9–1.8 g/L)	C4 (0.16–0.5 g/L)	Paraprotein			Follow up duration (Years)
									SPEP/SIFE	SFLC^b^ κ/λ (0.26–1.65)	Spot UPEP/UIFE	
1	CS/PE/CYC	No	41	62	90	Neg	1.08	0.18	Neg	0.95	Neg	1.5
2	CS/PE/CYC/AZA	No	24	159	30	Neg	0.93	0.25	Neg	1.29	Neg	2
3	CS/PE/RTX	Yes	165	168	38	Neg	0.84	< 0.03	IgM-κ (< 1 g/L)	1.47	Neg	5
4	CS/CYC/RTX/MS	No	302	106	51	Neg	1.55	< 0.03	Neg	2.09	Neg	11

*PCR* protein creatinine ratio; *eGFR* estimated glomerular filtration rate; *SPEP/SIFE* serum protein electrophoresis/immunofixation; *SFLC* serum free light chains; *κ* kappa; *λ* lambda; *CS* corticosteroids; *PE* plasma exchange; *CYC* cyclophosphamide; *AZA* azathioprine; *RTX* rituximab; *MS* mycophenolate sodium

^a^ MDRD

^b^ Freelite, UK

## Discussion and conclusions

We report four patients with noninfectious mixed CGN in whom MGUS was diagnosed using conventional methods for paraprotein detection [16, 26]. One in every five patients assessed in our cohort of noninfectious mixed CGN was found to have MGUS, although the true incidence of any such association remains uncertain owing to a paucity of data in the major published series [11, 27, 28]. This is partly because of limited biochemical analysis in previous studies, which have focussed exclusively on immunofixation of the cryoprecipitate. Whilst this remains a highly sensitive technique for detecting circulating mIg (> 0.05 g/L) in patients with type 1 or 2 cryoglobulinaemia, for example in comparison to SPEP (> 0.5 g/L) [29], its role in diagnosis of MGUS is not established. Thus all 20 patients in one series of noninfectious mixed CGN were shown to have type 2 CGN, with monoclonal gammopathy reported in 18 patients, yet without reference to cryoglobulin quantitation, SPEP, SIFE, SFLC, UPEP or UIFE [28]. These data were also not available in a recent series of 80 patients with noninfectious mixed CGN comprising 75 patients with type 2 CGN [11].

Conditions other than MGUS could potentially account for the development of mixed CGN in our cohort. pSS, which represents the commonest cause of mixed cryoglobulinaemia/CGN after HCV infection [8, 9, 11, 27, 28], was present in three of our four patients (conforming to current EULAR/ACR criteria [30]), including both cases of confirmed type 2 CGN. pSS involves a disease process of continuous polyclonal B cell activation, with malignant transformation of B cell clones in some cases, based on the increased incidence of lymphoma in patients with pSS [31, 32] (especially those with mixed cryoglobulinaemia [33, 34]). Of note, MGUS may also be more common in patients with pSS, and may confer an increased risk of developing lymphoma [35, 36] or myeloma [37]. It is therefore surprising that, prior to our study, coexistence of pSS and MGUS has not been reported in noninfectious mixed CGN. The additional finding in one of these patients of cholangiocarcinoma is also novel, although other solid organ malignancies have previously been reported in patients with noninfectious mixed CGN [38]. The one patient in our cohort who was confirmed as having type 3 CGN and who did not have pSS (Patient 3), was one of three cases in which only a very small monoclonal band (< 1 g/L) was identified. These observations raise important questions regarding the underlying pathophysiological processes responsible for noninfectious mixed CGN in the setting of multiple possible aetiologies, and the need for robust biochemical analysis as part of future studies, before causation should be attributed principally to MGUS.

It remains unclear from our study whether the identification of MGUS in a patient with noninfectious mixed CGN provides any guide to treatment and prognosis. A designation of MGRS implies the need for clonally targeted therapies to be considered, with the primary aim of improving renal outcomes [24]. Evidence for anticlonal therapy in patients with noninfectious mixed CGN consists largely of retrospective studies of rituximab, mostly in patients with type 2 CGN involving a monoclonal IgM-κ component [11, 27, 28, 39]. As in previous series of noninfectious mixed CGN [11, 27], our patients received multiple therapies including rituximab but also conventional immunosuppressive agents [11, 27]. Outcomes were generally favourable, and of the two patients with chronic kidney disease stage 3B at last follow-up, both had shown significant (25–40%) interstitial fibrosis on pre-treatment biopsies (potentially due to pSS-associated interstitial nephritis in one of these cases). Given previously reported, larger cohorts of noninfectious mixed CGN showing ESKD rates of 9–10% at 4 years [11, 28], our study possibly indicates that MGUS does not always confer a treatment-resistant course. No patient in our series received bortezomib, for which a single instance of use in refractory noninfectious mixed CGN is reported, in a patient with type 2 CGN and a monoclonal IgM-κ component, but no detectable monoclonal band on SPEP [40].

In conclusion, our study shows conclusively that MGUS may be present in a subset of patients with noninfectious mixed CGN. Even where this is the case, however, a designation of MGRS for cases of type 2 CGN may be open to question, given the observation of potential aetiologies for noninfectious mixed CGN other than MGUS.

## Data Availability

All data generated or analysed during this study are included in this published article.

## Abbreviations

CGN: Cryoglobulinaemic glomerulonephritis
MGRS: Monoclonal gammopathy of renal significance
mIg: Monoclonal immunoglobulin
SPEP: Serum protein electrophoresis
MGUS: Monoclonal gammopathy of undetermined significance
HCV: Hepatitis C virus
HBV: Hepatitis B virus
HIV: Human immunodeficiency virus
pSS: Primary Sjögren’s syndrome
SIFE: Serum Immunofixation
SFLC: Serum free light chains
UPEP: Urine protein electrophoresis
UIFE: Urine immunofixation
